# Strong coupling between surface plasmon polaritons and Sulforhodamine 101 dye

**DOI:** 10.1186/1556-276X-7-191

**Published:** 2012-03-19

**Authors:** Svitlana V Baieva, Tommi K Hakala, Jussi J Toppari

**Affiliations:** 1 Nanoscience Center, Department of Physics, P.O. Box 35, FI-40014, University of Jyväskylä, Finland; 2 School of Engineering and Applied Sciences, Harvard University, Cambridge, MA, 02138, USA

**Keywords:** surface plasmon polariton, Sulforhodamine 101, strong coupling, Rabi splitting, dispersion curve

## Abstract

We demonstrate a strong coupling between surface plasmon polaritons and Sulforhodamine 101 dye molecules. Dispersion curves for surface plasmon polaritons on samples with a thin layer of silver covered with Sulforhodamine 101 molecules embedded in SU-8 polymer are obtained experimentally by reflectometry measurements and compared to the dispersion of samples without molecules. Clear Rabi splittings, with energies up to 360 and 190 meV, are observed at the positions of the dye absorption maxima. The split energies are dependent on the number of Sulforhodamine 101 molecules involved in the coupling process. Transfer matrix and coupled oscillator methods are used to model the studied multilayer structures with a great agreement with the experiments. Detection of the scattered radiation after the propagation provides another way to obtain the dispersion relation of the surface plasmon polaritons and, thus, provides insight into dynamics of the surface plasmon polariton/dye interaction, beyond the refrectometry measurements.

**PACS: **42.50.Hz, 33.80.-b, 78.67.-n

## Background

The vast development of nanotechnology has mainly concentrated on the areas of novel materials or nanometer scale devices with electrical or (bio)chemical functionalities, while the optics and photonics have lacked behind, mostly due to the diffraction limit. While optical microcavities and photonic crystals have pushed the light to its spatial limit, already producing many interesting nonlinear effects [1], different methods are needed for a real nanoscale confinement. Many kinds of surface waves [2], e.g., surface phonon polaritons, surface magnetoplasmons, and, especially, surface plasmon polaritons (SPPs) [3,4], exist within the optical range and have been shown to provide the way for a possible nanoscale integration of photonics.

The surface plasmon polaritons are coupled modes of electromagnetic waves and oscillations of free electrons in a metal surface. They propagate in a wavelike fashion, like two-dimensional light bound to a metal-dielectric interface, however, with all the properties modified by the subwavelength confinement of these optical fully evanescent fields [3,4]. Therefore, SPPs offer fascinating prospects for the photoelectronics, for example, by evading the diffraction limit and, thus, enabling the efficient integration with electronics, so far prevented by the size mismatch of the ever diminishing electrical but diffraction limited optical components. In addition, a huge field enhancement near the interface, produced by the confinement, has been widely utilized in a surface enhanced Raman spectroscopy [5]. Similarly, also the enhancement of the fluorescence by surface plasmons has been under intense study [6,7]. Due to this, the combination of SPPs with emitters, such as dye molecules and quantum dots, is a heavily studied field of research nowadays. In many cases, the interactions between the SPPs and the emitters are governed by the weak coupling [6-12], resulting in a development of new nanodimensional photonic elements such as planar frequency converters [8] or planar refractive elements with a desirable refractive index [9]. Conversion from light to the SPP modes and vice versa can also be done by employing fluorescent molecules [10,11], and one of the most powerful techniques of the SPPs propagation imaging is also based on the scattering of the SPPs into photons that excite fluorescent molecules (or direct excitation of the molecules by SPPs) [12].

In a strong coupling regime, the interaction between the SPPs and the emitters cannot anymore be explained by the regular absorption and emission, based on the Fermi Golden rule. This gives rise to new interesting phenomena such as Rabi splitting. Vacuum Rabi splitting has been shown in, for example, microcavities [13-15] and in plasmonic systems with the SPPs coupled to J-aggregates [16,17], rhodamine molecules [18-20], and quantum dots [21,22]. In this regime, the Rabi splitting appearing in the dispersion relation of the SPP-exciton interaction is very similar to the one in the in-plane dispersion of the exciton-cavity photon interaction studied in detail. However, to achieve the SPP-exciton strong coupling no optical cavity is needed which makes the experiment much more practical to realize. Also, a large exciton linewidth of the usual dye molecules that is due to vibronic states and inhomogeneous broadening complicates the observation of the strong coupling regime in the quantum microcavities, but not in the case of SPPs [14,18-20].

In this article, we report on experiments showing the strong coupling between SPPs and Sulforhodamine 101 (SR101), which is an organic semiconductor that has two absorption maxima at around 550 and 600 nm^a ^and large oscillator strength. The samples consisted of a thin silver layer evaporated on the glass substrate, and a homogeneous organic semiconductor layer formed on the top of that. The excitation of SPPs was done by prism coupling technique (Kretschmann configuration) and Rabi splittings, with energies up to 360 and 190 meV, were obtained. In consequence of the observed double Rabi splitting, three separate SPP-exciton hybrid modes were formed, i.e., there exist three different possible frequencies for each wave vector; contrary to the case of periodically nonhomogeneous dielectric layers where several wave vectors exist for a single SPP frequency [23]. Due to the similarity to the exciton-cavity photon coupling, the formalism of the vacuum Rabi splitting [24] is easily adapted to explain the SPP dispersion relation. We use both the coupled oscillator model [17,18] and the transfer matrix method [25] to fit the experimental results. Finally, the scattered radiation of the propagating strongly coupled SPP-exciton hybrids was used for studying the dynamics and mode emission after the incoupling. Because of the propagation of the hybrid polaritons before scattering, the scattering event is spatially and temporally separated from the incoupling.

## Methods

### Sample details

Samples were fabricated on top of microscope cover glasses (colorless borosilicate glass, D263) with dimensions of 15 mm × 15 mm, and thickness ≈ 0.15 mm, purchased from *Knittel Gläser *(Waldemar Knittel Glasbearbeitungs, GmbH, Germany). Before fabrication, the glasses were thoroughly cleaned with hot acetone, followed by a sonication (5 min) in isopropyl alcohol and drying by dry nitrogen flow. After that approximately 55 nm thick silver layer was formed on top of the glass by electron-beam evaporation in an ultra-high vacuum (10^-9^-10^-8 ^mbar). It should be noted that the size of the Ag grains is dependent on the evaporation rate. There exists contradicting information in literature about the optimal evaporation rates of Ag layers for optical studies: whether to use a really high [26] or extremely slow rate [27] to obtain the lowest roughness. At the present work, the low evaporation rate of 0.02-0.04 nm/s was chosen to obtain smoother surface (2.5 nm RMS). Since all the samples reported in this study were prepared in different evaporation sessions, each time, the silver thickness and quality were verified by atomic force microscope.

**Figure 1:**
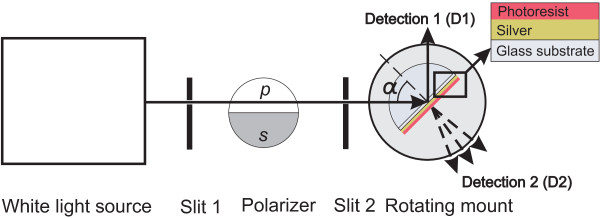
**Schematics of the experiment**. Schematic representation of the experimental setup and the sample structure. α is the angle of incidence.

Altogether, six samples with different SR101 concentrations were prepared. SU-8 epoxy-based negative polymer resist (Microchem SU-8 2025, MicroChem Corp., Newton, MA, USA) was used as a matrix for the molecules. Desirable amount of SR101 (Sigma-Aldrich, München, Germany) was firstly dissolved in 300 μl ethanol. We prepared samples that contained 0.3, 0.5, 1, 2, 3, and 4 mg SR101 and a reference sample without SR101. After that the obtained solution were mixed with 4.5 ml of cyclopentanone and 420 μl of already diluted SU-8 solution (1:6 volume ratio). It should be noted that in the solutions containing more than 3 mg of SR101, colloidal particles are formed. Further filtering was used to remove aggregates with the size above 0.2 μm. After the filtration, the resist was spin coated on top of the silver as also shown in Figure 1 and baked at 95°C. To obtain resist thickness of approximately 55 nm, the spinning rate of 4, 500 rpm was used for 45 s. The resist layer also protects the silver against the oxidation process. For the absorption measurement, reference samples without silver were similarly fabricated. The absorbance spectra were measured by Perkin Elmer UV/VIS Lambda 850 spectrometer (Waltham, MA, USA).

This fabrication routine produces samples with well reproducible characterristics. The SPP dispersion relation is strongly dependent on thicknesses of the silver and the dielectric layers, and silver quality, in addition to the dye molecule concentration. In order to compare the dispersion relations of samples with different dye concentrations, we kept all the other parameters unchanged during the fabrication. Yet, the standard microelectronics facilities utilized in the fabrication allow the easy integration and possible mass production of such devices.

### SPP excitation and detection methods

Figure 1 shows a schematic picture of our experimental setup. The hemicylindrical prism (ThorLabs, Göteborg, Sweden) made of BK7 glass with index of refraction of 1.52 was used in Kretschmann configuration [4,28,29]. The sample was installed on the flat face of the prism by index matching oil with the same refractive index, and Oriel 66182 white light source (Oriel Instruments, Stratford, CT, USA) was used for excitation. The light is collimated and aligned by two slits with rotatable Glan Taylor prism polarizer in between to adjust the polarization. The incident angle of the incoming light is adjusted manually by rotating the goniometric prism mount. The reflected (D1) and scattered (D2) signals (see Figure 1) are collected by an optic fiber connected to Jobin Yvon iHR320 spectrometer (HORIBA Jobin Yvon S.A.S., Longjumeau Cedex, France) equipped with Jobin Yvon Symphony CCD camera. It should be noted that the reflected and scattered signals were not collected simultaneously.

**Figure 2:**
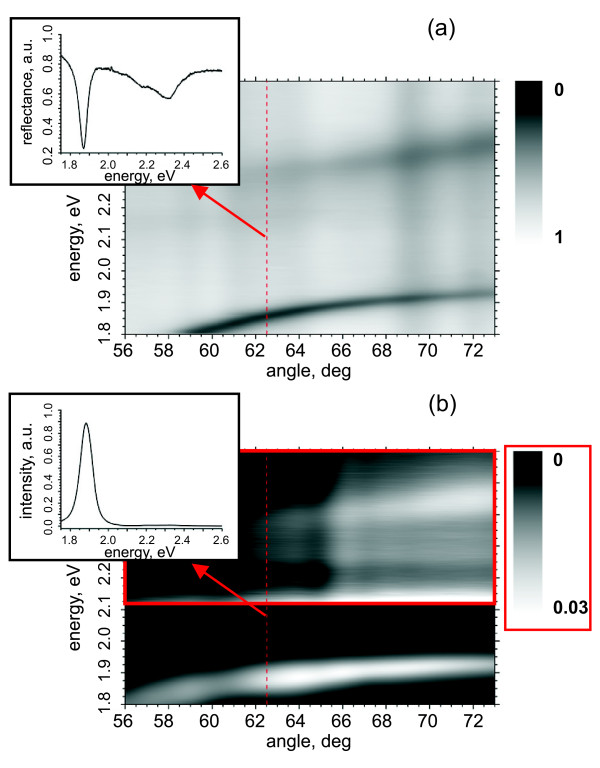
**Typical data obtained**. (**a**) Measured reflectance, D1, (2 mg SR101) as a function of the radiation energy and the angle of incidence. Inset shows the reflectance as a function of energy at the 63° excitation angle (red dashed line). (**b**) Normalized intensity of D2 signal from the same sample. Due to the weakness of the high energy peaks at D2, we zoom on the high energy region (indicated as green rectangle). Corresponding scale bar is presented within green rectangle. Inset shows the intensity as a function of the energy at the 63° excitation angle (red dashed line).

The typical data obtained by D1 and D2 are shown in Figure 2. The reflected light spectrum (D1) was collected for every angle of incidence higher than the total internal reflection angle and divided by the spectrum collected at 90° angle of incidence bypassing the prism and the sample. Then, the data were normalized to the highest value over the whole range. The data were collected at D2 with the same angles, but only normalized to the highest value over the whole range.

### Transfer matrix method

**Figure 3:**
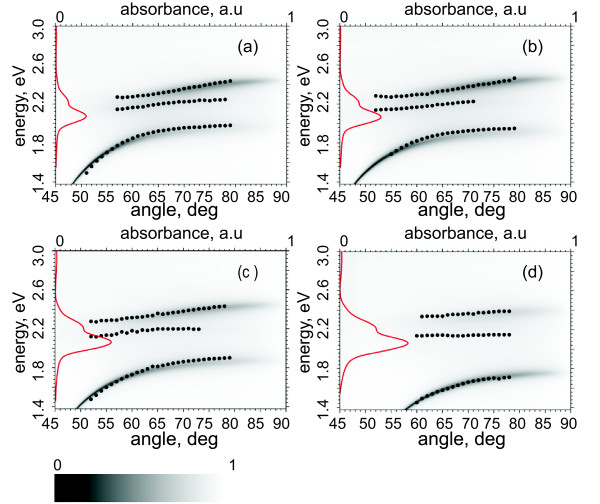
**Reflectance measurements and the transfer matrix theory compared**. Experimentally obtained energy dependences of the observed reflectance spectrum minima as a function of the angle of incidence (black dots). Calculated reflectance coefficients as a function of the excitation energy and the angle of incidence for 1 mg (**a**), 2 mg (**b**), 3 mg (**c**), and 4 mg (**d**) samples. Red line is the measured absorbance from the corresponding reference sample without silver as a function of excitation energy (scale on the top axis).

We analyzed the experimental data using two approaches: the transfer matrix method [25] and the coupled oscillators model. First one is simply based on the Fresnel equations for multilayered structures. The model system is a multilayered structure that consists of a semi-infinite dispersionless glass (dielectric constant *ε_g_*), 55 nm silver layer with the dielectric function calculated using the data from [26] 50 nm of resist containing SR101 and a semi-infinite air layer. The dielectric function of SR101 layer can be described by an equation [30]

(1) $$\varepsilon \left(\omega \right)=\varepsilon_{S}+\overset{2}{\underset{i=1}{\sum }}\frac{A_{i}\omega_{0i}^{2}}{\omega_{0i}^{2}-\omega^{2}-i\omega \gamma_{i}},$$

where *ε_S _*is a frequency independent dielectric permittivity of the media that is hosting the SR101 molecule (SU-8 in here). *A_i _*is a dimensionless parameter characterizing the strength of an oscillation with the resonance frequency *ω*_0*i*_, and *γ_i _*describes damping of such an oscillation. With SR101, *i *= 1, 2. We deduced the parameters *A_i_, ω*_0*i*_, and *γ_i _*from the measured absorption spectra of the reference samples containing only the SR101 resist spun on the glass substrate. The list of the parameters used in the fitting are presented in Table 1. Figure 3 shows the calculated reflectance coefficient of the model system together with the experimental data.

**Table 1:** Parameters of the transfer matrix theory.

Sample (mg)	*ε_s_*	*ε_g_*^b^	*A*_1_	*A*_2_	*γ*_1_, eV	*γ*_2_, eV	*ω*_01_, eV	*ω*_02_, eV
1	2.9	1.485	0.094	0.0065	0.2	0.11	2.06	2.25
2	3	1.505	0.133	0.013	0.2	0.12	2.05	2.23
3	3.1	1.485	0.168	0.019	0.18	0.11	2.05	2.22
4	3	1.33	0.226	0.029	0.2	0.14	2.05	2.23

^b^We allow changes in this parameter to compensate a small uncertainty in the determination of the angle of incidence due to possible not perfect alignment of the prism optical axis and the axis of rotation, and the small deviation from the ideal hemicylindrical shape.

It is important to realize that D2 data result from the SPP scattering on silver film imperfections, and it is not the transmittance in terms of the transfer matrix method. D2 signal is also affected by the SPP/SR101 interaction that is beyond the model described above. So the D2 data cannot be fitted with the transfer matrix method directly.

### Dispersion curves and the coupled oscillators model

To obtain the energies for the Rabi splits, we need the dispersion relations, i.e., energy dependence of the mode on the in-plane wave vector component *k_x_*. The dispersion curves can be achieved by fitting Lorentzian curves to the dips in the reflectance (D1, Figures 1 and 2a) or peaks of the spectra resulting from the SPPs scattering to photons (D2, Figures 1 and 2b). We are able to resolve all the three hybrid branches in both cases even though the high energy branches are very weak in D2 (see Figures 2 and 3).

The coupled oscillators model is employed to fit the dispersion relation [17,18]. In this model, we solve a system of coupled equations for the interacting SPP and the two SR101 excitons that correspond to the absorption minimum and shoulder,

(2) $$\left(\begin{array}{ccc} E_{SPP}\left(\text{Re}\;\left[k_{x}\right]\right)-i\gamma_{SPP} & V_{1} & V_{2}\\ V_{1} & E_{Ex1}-i\gamma_{Ex1} & 0\\ V_{2} & 0 & E_{Ex2}-i\gamma_{Ex2} \end{array}\right)\left(\begin{array}{c} x_{SPP}\\ x_{Ex1}\\ x_{Ex2} \end{array}\right)=\varepsilon \left(\begin{array}{c} x_{SPP}\\ x_{Ex2}\\ x_{Ex3} \end{array}\right)$$

**Figure 4:**
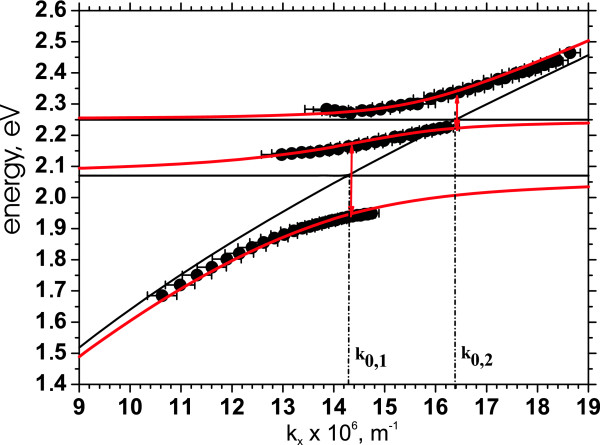
**Determining the Rabi energies by the coupled oscillator model**. Dispersion relation of the sample having 2 mg SR101 (solid black circles) fitted with the coupled oscillator model (solid red). Black solid lines represent the solution of the Equation 2 with zero couplings (*V*_1_, *V*_2 _= 0), and the black dashed lines indicate the wave vector values at which the anti-crossings occur. Red vertical arrows indicate the Rabi energies.

Here, *E_SPP_*(Re[*k_x_*]) is a *k_x_*-dependent energy of the uncoupled SPP at the interface between the silver layer and SU-8 (no SR101), and *γ_SPP _*is the linewidth of the SPP. *E_Exi _*and *γ_Exi _*are the energy and the linewidth of the *i *th exciton, respectively, and *V_i_*is the coupling strength of that with the SPP (*i *= 1, 2). *x_SPP_, x*_*Ex*1_, and *x*_*Ex*2 _are the state vector components corresponding to the uncoupled SPP and the excitons, respectively. For simplicity, we follow the common way and neglect the linewidths of the excitations. The coupling strength parameters are varied to maximize the overlap with the experimental data. The values of the energy splits are subsequently deduced from the fitted data as shown in Figure 4.

## Results and discussion

### Detection 1 (D1)

**Figure 5:**
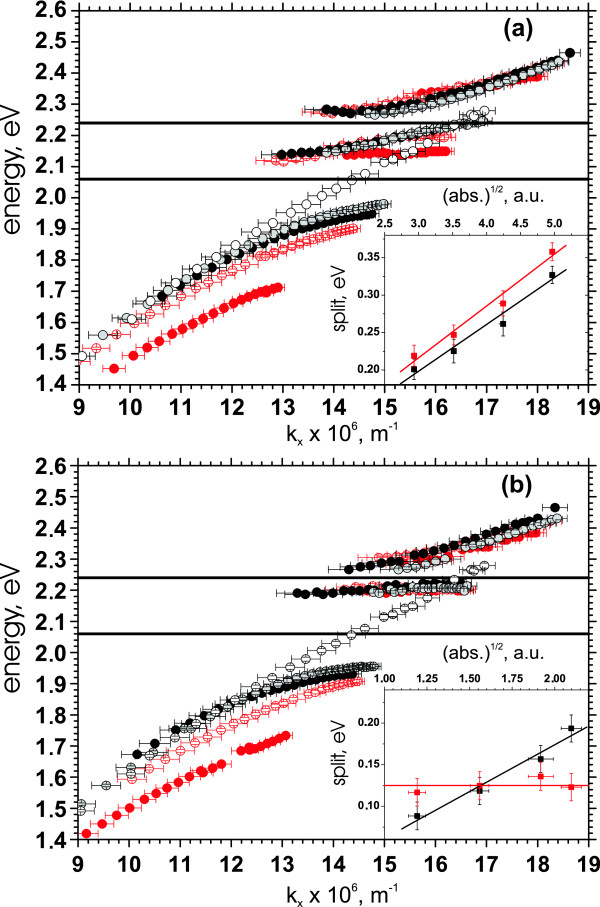
**Measured dispersion curves and the Rabi energies**. The dispersion curves of the four samples having 1 mg SR101 (solid gray circles), 2 mg (solid black circles), 3 mg (empty red circles), and 4 mg (solid red circles) measured by D1 (**a**) and D2 (**b**). Empty black circles represent the dispersion relation for the sample without SR101 molecule measured at D1. Solid black horizontal lines are the SR101 absorption maximum and the absorption shoulder energies. The insets show the low (**a**) and high (**b**) energy splits as a function of (absorbance)^1/2 ^with linear fits. Black dots correspond to D1, and red dots correspond to D2. Each curve corresponds to a single measured sample, and the error bars represent the estimated uncertainty of the measurement instruments (goniometric tool scale reading and wavelength determination).

Dispersion curves measured by the method D1 from four samples are represented on Figure 5a. The behavior of the dispersion relation with changing dye concentration is in good agreement with the previously reported observations [17,18], and the theories as shown in Figures 3 and 4. In the case of D1, the sample without SR101 has a continuous dispersion curve without any signatures of splitting as seen on Figure 5a. When SR101 is present, the interaction between SPP and the molecular excitons happens, and an anti-crossing behavior is observed. For low concentration samples (less than 1 mg of SR101), only the lower energy gap, corresponding to the absorption maximum, opens. By increasing the concentration, this gap widens, and the second gap, corresponding to the absorption shoulder, appears. If the concentration increases, more both splits widen as seen on Figures 3 and 5a. Thus, by adjusting the concentration of the molecules in the film, one can move over from a weak coupling regime to the strong coupling regime.

The width of the energy gap should be linearly dependent on the square root of the effective oscillator strength or, in other words, the total absorbance [18,31]. For D1, the lower and the upper energy gap values behave according to this prediction (insets on Figure 5). We find that the lower energy gap value changes from 200 to 330 meV when the amount of SR101 changes from 1 to 4 mg. These values well exceed the split energies reported so far in references 15-18. The upper energy gap values vary from 90 to 190 meV.

### Detection 2 (D2)

The same behavior characterizes the dispersions obtained from the scattered light detection, i.e., D2. However, the data from D2 shows larger values for the lower energy gap in comparison to D1, even the linear dependence on the square root of the total absorbance still holds as shown in the inset on Figure 5a. The lower gap value now changes from 220 to 360 meV. The increase of the lower energy gap in D2 agrees well with our earlier measurements and can be understood in the following way demonstrated in the reference 18. Since the interaction of the molecules with the SPP is coherent, and the experiment is within a vacuum Rabi split regime, as shown by the intensity independency of the splits, the molecules taking part to the coupling with a single SPP can be considered as a single high strength oscillator. As already noted and shown, the number of molecules taking part in the strong coupling is proportional to the concentration of molecules, i.e., the reference sample absorbance. But while comparing the D1 and D2 measurements, there is no difference in the concentration. However, in the case of reflectance, D1, the photon/SPP has a limited interaction time with molecules until reflected back to the detector. During this time, the SPP does not reach its full spatial coverage determined by the propagation speed and the decoherence time and, thus, only interacts with a limited number of molecules making the oscillator strength effectively smaller. However, in D2, we detect scattered SPPs, which have propagated along the silver/SR101 resin boundary, and the spatial coverage of the SPP has reached its full value, thus, enabling more dye molecules to be involved in the interaction process, which further increases the effective oscillator strength. In the other words, the interaction time has, thus, also increased, which similarly increases the gap as will be discussed in more details on the next chapter. Yet, our observation shows that approximately 10% more molecules participate in decoupling to photons compared to the incoupling.

### Further discussion

However, contradicting the earlier results and the reasoning in reference 18 and above, the upper energy gap is independent on the sample's absorbance and stays close to 125 meV in the case of D2. This also implies that with high SR101 concentrations, i.e., for the samples with more than 2 mg of SR101, the gap energy is smaller in D2 than in D1, since in D1, the gap depends on the absorption as shown in the inset in Figure 5b. This tendency of the upper energy gap to stay constant in D2 can find the explanation via considering the role of the SPP lifetime that is proportional to (*γ_SPP_*)^-1^. In simpler case, when SPP interacts with only one exciton, the energy gap value can be easily written as

(3) $$\Delta E=\sqrt{4V^{2}-{\left(\gamma_{SPP}-\gamma_{Ex}\right)}^{2}}$$

The term *V *∝ (*absorbance*)^1/2 ^is responsible for the growth of the gap when more molecules are involved in the coupling, i.e., increase in the concentration or via propagation. However, if considering *γ_Ex _*as a constant (well justified approximation in the case of molecular excitations), the increase of *γ_SPP_*, i.e., the decrease of the SPP lifetime, can lead to a decrease of the energy split (in uncoupled case *γ_SPP _*≈ *γ_Ex_*). Even though for SPP interacting with two excitons, the energy split dependence on the couplings strengths and the SPP lifetime is more complex than the Equation 3, and the change in SPP lifetime can compensate the gap growth due to the increased oscillator strength.

One possible reason for the decrease of the SPP lifetime would be an exciton-phonon scattering, which can cause population transfer from the upper polariton branches to the lowest one, or faster decay of the upper branches via other channels, both characterized by a decreased luminescence of the upper branches compared to the lowest one, i.e., D2 in our case [32]. This signature is clearly visible in our measurements also, as evidenced by comparing the D1 and D2 signals in the Figure 2. In addition, the same was observed in our earlier measurements [18], where an energy transfer was also suggested to be the reason. In addition to the decreased luminescence, these scattering processes would significantly shorten the lifetime of the upper-branch-polaritons leading to the decrease of the Rabi energy of the higher split in D2, especially in the case of SR101, where larger couplings are achieved compared to the earlier measurements by Rhodamine 6 G [18,19]. Further, the strength of these processes is proportional to the coupling strength [32], which means that by increasing the concentration of SR101, the lifetime of the upper-branch-polaritons gets shorter (visible also as a widening of the linewidth of the upper branches in Figure 3 as the concentration increases). This furthermore decreases the Rabi split via increasing *γ_SPP _*and could compensate the increase in *V *also resulting from the increased SR101 concentration. The same does not hold for the lower split since the polaritons are gathered to the lowest branch involved in that.

## Conclusions

We have experimentally demonstrated the strong coupling between SPPs and SR101 dyes in the planar silver/SR101 resin structures by using two complementary detection geometries. Double Rabi splitting was observed with the energies higher than in the earlier measurements, i.e., up to 360 and 190 meV. Both, the transfer matrix method and the coupled oscillator models fit the experimental data nicely, and the energies of the observed Rabi splittings depend linearly on the square root of the oscillator strength proving the coherent coupling between the SPP and the dye molecules. The reflectrometry measurements (D1) show that the lower and the upper gap values are changed by 100 meV while increasing the molecule concentration four times. The employed scattered radiation measurements (D2) allowed us gaining insight into the dynamics of the SPP/molecule interaction. The lower energy gap values obtained by the D2 measurements are approximately 10% larger than the gaps in D1, which is consistent with the earlier observations and means that more dye molecules participate in the decoupling to light than the initial incoupling. However, the upper energy split is most probably influenced by a strong decrease of the SPP lifetime leading to a decrease of the Rabi energy in D2.

## Abbreviations

SPP: surface plasmon polariton; SR101: Sulforhodamine 101; D1: detection method 1; D2: detection method 2.

## Competing interests

The authors declare that they have no competing interests.

## Authors' contributions

TKH and JJT initiated the idea. SVB carried out the sample fabrication, experiments, and analysis and wrote the first version of the manuscript. TKH and SVB built the experimental setup. JJT was involved in the design, coordination, and analysis of the experiments, and revising of the manuscript for improving the concepts. All authors read and approved the final manuscript.

## Endnotes

^a^Measured absorption maxima of 50 nm thick SR101/SU-8 film deposited on a glass substrate.
